# Rapid fabrication of ultra-thin 2D metal–organic framework membranes for accurate gas separation

**DOI:** 10.1093/nsr/nwaf301

**Published:** 2025-07-29

**Authors:** Chenyu Zhu, Yuan Peng, Kun Li, Wentai Hu, Weishen Yang

**Affiliations:** State Key Laboratory of Catalysis, Dalian Institute of Chemical Physics, Chinese Academy of Sciences, China; University of Chinese Academy of Sciences, China; State Key Laboratory of Catalysis, Dalian Institute of Chemical Physics, Chinese Academy of Sciences, China; University of Chinese Academy of Sciences, China; State Key Laboratory of Catalysis, Dalian Institute of Chemical Physics, Chinese Academy of Sciences, China; University of Chinese Academy of Sciences, China; State Key Laboratory of Catalysis, Dalian Institute of Chemical Physics, Chinese Academy of Sciences, China; University of Chinese Academy of Sciences, China; State Key Laboratory of Catalysis, Dalian Institute of Chemical Physics, Chinese Academy of Sciences, China; University of Chinese Academy of Sciences, China

## Abstract

A high throughput synthesis method is established to produce ultrathin MOF nanosheets and nanosheet membranes within only 30 min, shedding light on 2D high-performance membrane customization for separation and purification requirements.

Two-dimensional metal–organic framework (2D MOF) molecular sieve membranes stand out due to three key advantages: tunable porous architectures that allow structural customization, ultra-high pore density that enables precise molecular discrimination and nanometer-scale thickness that minimizes mass transfer resistance [1]. These attributes make 2D MOF membranes ideal candidates for achieving both high selectivity and high permeance, which can significantly reduce overall separation costs and improve process efficiency. Such membranes are especially promising for challenging separations involving molecules of similar sizes, such as H_2_ purification and CO_2_ capture from steam reforming off-gas in industrial H_2_ production (kinetic diameters: H_2_ ∼0.29 nm, CO_2_ ∼0.33 nm). However, fabrication methods play a critical role in realizing the performance potential of 2D MOF membranes. Conventional approaches have limitations: layer-by-layer assembly of exfoliated nanosheets is time-consuming [2] and modulator-directed crystallization in bulk solution often requires extra structure-directing agents to enforce a 2D structure [3]. In short, current fabrication techniques for 2D MOF membranes remain inefficient (Table S1).

In this work, we developed a one-pot air–water interfacial synthesis strategy to rapidly fabricate ultra-thin 2D MOF molecular sieve membranes (Fig. 1a and Fig. S1). As a proof of concept, we employed a reaction system comprising a zinc source and hydrophobic benzimidazole (BIM) ligand. Scanning electron microscopy (SEM) and transmission electron microscopy (TEM) confirmed the formation of discrete MOF nanosheets at the interface. The resulting 2D nanosheets exhibited a few-layered morphology with lateral dimensions in the order of microns (Fig. 1b and Fig. S2). Atomic force microscopy (AFM) revealed an ultra-thin thickness of ∼7.7 nm for the nanosheets (Fig. 1c and inset), corresponding to an extremely high aspect ratio of ∼10 000. This result highlights the effectiveness of our synthesis strategy for producing large-area, ultra-thin sheets. Notably, the nanosheets adhered closely to both hydrophobic polyvinylidene fluoride (PVDF) and hydrophilic silicon wafer substrates without wrinkling (Fig. 1b and c), indicating strong affinity to substrate surfaces regardless of surface chemistry.

**Figure 1.**
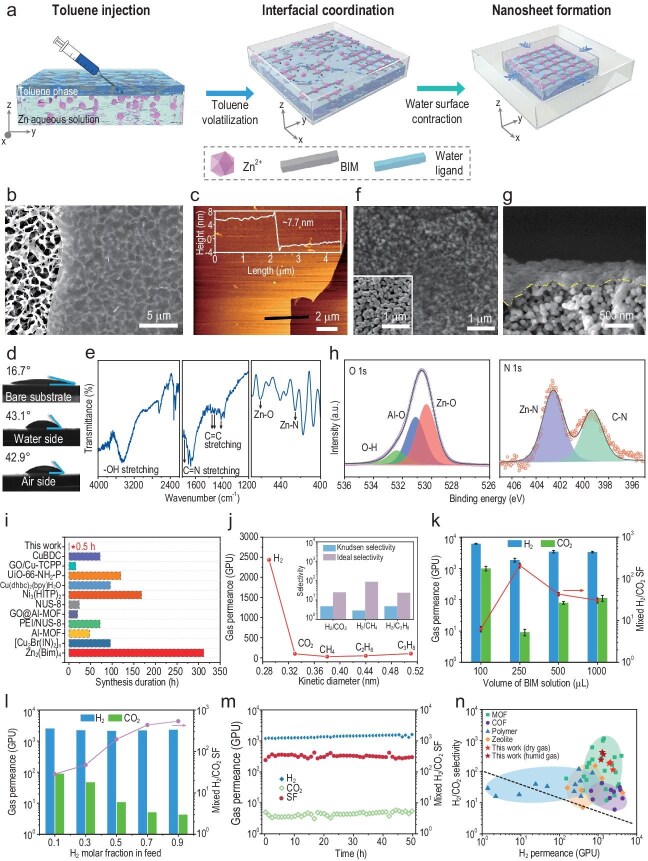
Ultra-thin MOF nanosheet membranes fabricated via rapid interfacial synthesis and their gas-separation performances. (a) Schematic illustration of air‒water interfacial synthesis and sequential water-level drop (surface constriction) process for MOF nanosheet membrane fabrication. (b) SEM and (c) AFM images of Zn–MOF nanosheets on PVDF and silicon wafer substrates, respectively. Height profile along the black line is shown in (c). (d) WCAs of a bare hydrophilic Al_2_O_3_ substrate, the air side and the water side of the Zn–MOF nanosheets supported on an Al_2_O_3_ substrate. (e) FTIR spectrum of Zn–MOF nanosheet sample. (f) Surface and (g) cross-sectional SEM images of the obtained Zn–MOF nanosheet membrane supported on a porous Al_2_O_3_ substrate. The inset in (f) shows the surface of a bare substrate for comparison. The dashed line in (g) highlights the outline of the fractured cross section of the ultra-thin membrane. (h) XPS spectra of N 1s and O 1s of the membrane. (i) Comparison of the synthesis durations of typical 2D MOF membranes reported to date (taking the minimum estimated time for each; see Table S1 for details). (j) Single-gas permeations through the Zn–MOF nanosheet membrane as a function of the molecular kinetic diameter. Inset: ideal and Knudsen selectivities for H_2_/CO_2_, H_2_/CH_4_ and H_2_/C_3_H_8_ gas pairs. (k) Effect of BIM ligand dosage on gas-separation performance of the Zn–MOF nanosheet membrane. (l) Effect of H_2_/CO_2_ feed molar ratios on the separation performance of the Zn–MOF nanosheet membrane. (m) Long-term stability of the Zn–MOF nanosheet membrane for H_2_/CO_2_ separation under 100% relative humidity at room temperature. (n) Comparison of H_2_/CO_2_-separation performance between the Zn–MOF nanosheet membranes and other reported membranes. The 2008 polymer upper bound is plotted assuming a membrane thickness of 0.1 μm and shown as a dashed line.

To further examine the in-plane structure of the nanosheets, we measured water contact angles (WCAs) on both sides of the obtained nanosheets. The WCA values were nearly identical on the two faces (Fig. 1d), indicating similar surface chemistry on each side. Fourier transform infrared (FTIR) spectroscopy confirmed the incorporation of organic ligands, with characteristic peaks at 1685 and 1407–1477 cm^−^¹ corresponding to C=N and C=C stretching vibrations, respectively (Fig. 1e). The formation of Zn–N coordination bonds is also evident from the peak at 425 cm^−^¹ [4]. Additionally, FTIR peaks at 3470 and 461 cm^−^¹ indicated the formation of Zn–O bonds in the framework, likely originating from water molecules present in the reaction medium [5]. These observations suggested that the Zn–MOF nanosheet likely has a mixed-ligand coordination framework in which Zn nodes are randomly coordinated by both BIM ligands and water molecules in-plane (Fig. S3) [6].

We found that increasing the reactant concentration facilitated a spontaneous ordered assembly process, which enhanced structure integrity and expanded the lateral dimensions of the resulting nanosheets (Fig. S4). Notably, ultra-thin and discrete nanosheets were effectively produced with as little as 250 μL of BIM solution (Fig. 1b and c, and Figs S2 and S4c). In contrast, excessive BIM amounts led to the formation of thicker, less transparent nanosheets (Fig. S4d and e), which were not conducive to constructing ultra-thin, compact membranes. At such a low BIM dosage, the ligand is rapidly consumed during interfacial synthesis, decreasing its local concentration and limiting further growth. To overcome this limitation, we introduced a controlled water–surface contraction step during synthesis. By gradually lowering the water level in a conical funnel (Fig. S1), a horizontal radial compression force was generated, which drew the nascent quasi-nanosheet ‘islands’ together and locally increased the BIM ligand concentration per unit area. This timely compression step is essential for the Zn–BIM oligomers to coalesce into micron-sized nanosheets. Otherwise, only amorphous or hole-riddled films were obtained (Fig. S5).

Another distinct merit of our synthesis strategy is the dramatically shortened synthesis time compared with conventional protocols that typically require several hours or even days (Table S1). In our case, just 30 minutes of reaction yielded a satin-like ultra-thin nanosheet film floating at the interface (Fig. S6). This dramatic reduction in fabrication time highlights the feasibility and scalability of the approach for practical separation applications. Remarkably, nanosheets with large folds, moderate mechanical strength and minor cracks could even form within as little as 10 minutes (Fig. S7). However, prolonging the reaction time without incorporating the timely water–surface contraction step led to poor-quality products. In such a case, Zn–BIM coordination oligomers failed to assemble into well-defined nanosheets and instead remained scattered around underdeveloped quasi-nanosheets (Fig. S6c and d). These findings reaffirm that combining an ultralow BIM ligand dosage with timely water–surface contraction is crucial for producing intact, ultra-thin MOF nanosheets in a rapid manner.

The other key challenge is the gentle and orderly assembly of nanosheet building blocks in parallel with a continuous, ultra-thin membrane. In our approach, water itself serves as a self-leveling platform that facilitates the orderly arrangement of nanosheets at the air–water interface [7]. Notably, we used the same water medium for both nanosheet growth and assembly, allowing seamless integration of these steps. After synthesis, gradual removal of water from the conical funnel induced additional surface contraction, prompting the nanosheets to gather into a compact layer. This floating layer was then carefully transferred onto a porous α-Al_2_O_3_ substrate by gently brushing the water surface (Fig. S1). Surface SEM imaging revealed a semitransparent, satin-like nanosheet layer uniformly covering the substrate, with the underlying Al_2_O_3_ grains still visible (Fig. 1f, inset). The nanosheet layer adhered closely to the rough substrate surface, driven by capillary forces from the substrate pores and the intrinsic flexibility of the MOF nanosheets. No pinholes, cracks or folds were observed in the membrane. Elemental mapping confirmed a uniform Zn–ligand coordination structure throughout the membrane (Fig. S8). A cross-sectional SEM image of fractured membrane highlighted its ultra-thin thickness (dashed outline in Fig. 1g), consistent with the ∼7.7-nm thickness of the individual nanosheet building blocks. X-ray photoelectron spectroscopy (XPS) further confirmed that the Zn–BIM-water coordination structure of the nanosheets was preserved in the membrane. The N 1s spectrum showed the presence of Zn–N bonds and the O 1s spectrum revealed Zn–O bonds (Fig. 1h) [8,9]. These results demonstrate the feasibility of seamlessly linking nanosheet formation and membrane assembly in an integrative water-based process. To our knowledge, very few studies have achieved MOF membranes with such low ligand consumption or in such a short synthesis time (Fig. 1i and Table S1).

The gas-separation performance of the ultra-thin Zn–MOF membrane was evaluated by using a Wicke–Kallenbach cell. Single-gas permeation tests showed a sharp cut-off between H_2_ and larger gas molecules (Fig. 1j), confirming a molecular sieving capability of the obtained membrane. The effective pore aperture of the Zn–MOF framework was estimated to be ∼0.3 nm [2]. Membranes fabricated with varying BIM ligand dosages exhibited a clear volcano-shaped trend in the H_2_/CO_2_ separation factor (SF) (Fig. 1k). The H_2_/CO_2_ mixture-separation performance was consistent with the single-gas results. The optimal BIM dosage of 250 μL produced a tightly stacked nanosheet configuration, achieving an impressive SF of 210 ± 28 and an exceptional H_2_ permeance of 1862 ± 252 GPU (1 GPU = 3.35 × 10^−10^ mol/m²·s·Pa at standard temperature and pressure). This combination of high selectivity and high permeance highlights the membrane's great potential for practical H_2_ purification and CO_2_-capture applications.

To gain insight into the separation mechanism, we tested H_2_/CO_2_ gas mixtures with different feed compositions. As the H_2_ molar fraction in the feed increased, the CO_2_ permeance significantly declined while the H_2_ permeance remained nearly constant (Fig. 1l). This trend strongly indicated that molecular sieving is the dominant separation mechanism. Conversely, at a higher CO_2_ molar fraction, the CO_2_ permeance increased, suggesting that the competitive adsorption of CO_2_ in the Zn–MOF pores contributed to transport under those conditions. These characteristics make the membrane particularly effective in H_2_-rich environments, such as those generated by methanol reforming. Additionally, we also examined the effect of temperature on separation performance (Fig. S9a). Both the H_2_ and CO_2_ permeances increased with temperature, corresponding to apparent activation energies (*E*_app_) of 4.3 kJ/mol for H_2_ and 10.2 kJ/mol for CO_2_ (Fig. S9b). These values align well with those reported for conventional microporous molecular sieve membranes [1,2], further reinforcing the dominance of a molecular sieving mechanism.

Long-term stability tests under harsh conditions (100% relative humidity at ambient temperature) demonstrated the robustness of the ultra-thin molecular sieve membrane. Over prolonged operation, the H_2_/CO_2_ SF stabilized at ∼300 and the H_2_ permeance remained at >1000 GPU (Fig. 1m). These results indicated that the membrane maintained excellent performance even in fully humidified streams, which is an important practical requirement. Notably, compared with other reported H_2_/CO_2_-separation membranes, the Zn–MOF molecular sieve nanosheet membranes, developed with superior efficiency and economy, far exceeded the 2008 upper bound and ranked among the top performers (Fig. 1n and Table S2).

Finally, we demonstrated the broad applicability of the air‒water interfacial synthesis strategy by extending it to a diverse set of MOF systems. Using 7 different metal precursors and 5 different organic ligands, we successfully synthesized 12 additional ultra-thin 2D MOF nanosheets (Fig. 2a and b, and Fig. S10). Each of these new nanosheets corresponded to 2D isomers of a known 3D MOF structure, confirming that our method is not limited to a single framework type. All of the obtained nanosheets had lateral dimensions in the order of microns and exhibited the characteristic flexibility of 2D nanosheets, underscoring the broad applicability of the fabrication strategy compared with other methods [10]. This ability to tune a wide range of metal–ligand combinations and pore architectures will greatly facilitate the customization of MOF nanosheets for targeted applications such as separation, catalysis and sensing.

**Figure 2.**
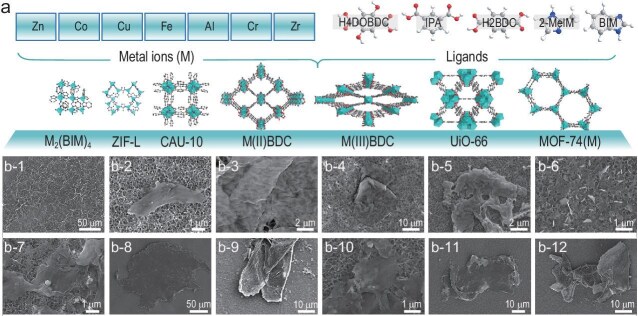
(a) Validation of the universality of the interfacial synthesis for various 2D MOF nanosheets. Seven different metal sources and five organic ligands were used to fabricate 2D isomers of the corresponding MOF structures. H_4_DOBDC: 2,5-dihydroxyterephthalic acid; IPA: isophthalic acid; H_2_BDC: terephthalic acid; 2-MeIM: 2-methylimidazole. Corresponding SEM images of the supposed 2D isomers of (b-1) Co_2_(BIM)_4_, (b-2) ZIF-L(Zn), (b-3) CAU-10, (b-4) ZnBDC, (b-5) FeBDC, (b-6) CuBDC, (b-7) CrBDC, (b-8) AlBDC, (b-9) MOF-74(Zn), (b-10) MOF-74(Cu), (b-11) MOF-74(Fe) and (b-12) UiO-66.

In conclusion, we have developed a convenient and rapid air–water interfacial synthesis strategy for fabricating ultra-thin 2D MOF molecular sieve membranes. This approach dramatically reduced the membrane preparation time from conventional hours or days to minutes and it produced MOF nanosheets with ultra-large aspect ratios while minimizing ligand consumption. Using Zn–MOF nanosheets as a demonstration, we constructed H_2_/CO_2_-separation membranes that ranked among the top performers reported to date. These results open up new avenues for the on-demand customization of MOF nanosheets and 2D MOF membranes to meet diverse high-performance separation needs.

## Supplementary Material

nwaf301_Supplemental_File
