# Supplementary material for: Rapid fabrication of ultra-thin 2D metal–organic framework membranes for accurate gas separation
Source: Natl Sci Rev. 2025 Jul 29;12(9):nwaf301. doi: 10.1093/nsr/nwaf301 (PMC12382440; doi:10.1093/nsr/nwaf301)
Supplement: nwaf301_Supplemental_File [file nwaf301_supplemental_file.pdf]

NSR National Science Review

Supplementary Information for

Rapid fabrication of ultra-thin 2D metal-organic framework membranes for accurate gas separation

Chenyu Zhu, Yuan Peng*, Kun Li, Wentai Hu, and Weishen Yang*

* **Correspondences.** E-mail: pengyuan@dicp.ac.cn, yangws@dicp.ac.cn

This PDF file includes:

Supplementary Materials and Methods
Supplementary Figures
Supplementary Tables
Supplementary References

Supplementary Materials and Methods

Materials

Zinc acetylacetonate hydrate ($Zn(acac)_2$, Sigma-Aldrich, ≥99%), zinc nitrate hexahydrate

($Zn(NO_3)\cdot 6H_2O$, Sigma-Aldrich, ≥99%), cobalt acetylacetonate ($Co(acac)_3$, Sigma-Aldrich, 98%), aluminum acetylacetonate ($Al(acac)_3$, Sigma-Aldrich, 98%), zirconium tetrachloride ($ZrCl_4$, Aladdin, 98%), ferric nitrate nonahydrate ($Fe(NO_3)_3\cdot 9H_2O$, Sigma-Aldrich, ≥98%), copper nitrate trihydrate ($Cu(NO_3)_2\cdot 3H_2O$, Sigma-Aldrich, ≥99%), aluminum nitrate nonahydrate ($Al(NO_3)_3\cdot 9H_2O$, Sigma-Aldrich, ≥98%), chromium nitrate nonahydrate ($Cr(NO_3)_3\cdot 9H_2O$, Sigma-Aldrich, 99%), benzimidazole (BIM, Sigma-Aldrich, 98%), 2-Methylimidazole (2-MeIM, Sigma-Aldrich, 99%), 2,5-Dihydroxyterephthalic acid (H4DOBDC, Sigma-Aldrich, 98%), isophthalic acid (IPA, Sigma-Aldrich, 99%), terephthalic acid (H2BDC, Sigma-Aldrich, 98%), toluene (Sinopharm Chemical Reagent Co., LTd, ≥99.5%). All of the chemicals were used without further purification. Deionized water was used throughout the experiments.

The substrates used in this work were asymmetric α-Al_2O_3 discs with a diameter of 18 mm and a thickness of 1 mm, which were purchased from Fraunhofer IKTS (Germany). The average aperture of the top layer is approximately 70 nm. All discs were cleaned twice with deionized water and once with acetone before use.

Preparation of 2D Zn-MOF nanosheets and nanosheet membranes

A solution of 131.8 mg $Zn(acac)_2$ in 250 mL water and 10 mg BIM in 10 mL toluene was prepared separately. After complete dissolution, 100 mL of the $Zn(acac)_2$ aqueous solution was added into a custom-made stainless-steel conical funnel with a needle valve. Then, a specific volume (50/100/250/500/1000 μL) of BIM in toluene was gently dropped onto the aqueous surface to form a stable air-water interface. Due to the rapid evaporation of toluene at room temperature, a BIM ligand layer formed spontaneously at the interface, enabling interfacial coordination with Zn^{2+} ions.

To prevent disturbance of the interface, the reaction proceeded undisturbed for a set duration (10/30/60/120 min). The funnel valve was adjusted to drain the water at approximately one drop per second, facilitating ultra-thin MOF nanosheets formation at the air-water interface (Figure S1). The nanosheets were collected and subjected to dialysis in deionized water with a dialysis tube (MW: 14000) for one week to remove residual reactants and oligomers prior to characterizations.

To demonstrate the generality of this fabrication strategy, 9 kinds of metal salts and 5 kinds of organic ligands were employed: $Co(acac)_3$ and BIM for $Co_2(BIM)_4$, $Zn(acac)_2$ and 2-MeIM for ZIF-L(Zn), $Al(acac)_3$ and IPA for CAU-10, $Zn(NO_3)\cdot 6H_2O$ and H2BDC for ZnBDC, $Fe(NO_3)_3\cdot 9H_2O$ and H2BDC for FeBDC, $Cu(NO_3)_2\cdot 3H_2O$ and H2BDC for CuBDC, $Cr(NO_3)_3\cdot 9H_2O$ and H2BDC for CrBDC, $Al(NO_3)_3\cdot 9H_2O$ and H2BDC for AlBDC, $Zn(acac)_2$

and H4DOBDC for MOF-74(Zn), $Cu(NO_3)_2\cdot 3H_2O$ and H4DOBDC for MOF-74(Cu), $Fe(NO_3)_3\cdot 9H_2O$ and H4DOBDC for MOF-74(Fe), $ZrCl_4$ and H2BDC for UiO-66.All followed the same protocol as described above for Zn-MOF nanosheets.

To fabricate membranes, the water level was further lowered in the same custom-made stainless-steel conical funnel, compressing the nanosheets into a dense layer on the water surface. An α-Al_2O_3 disc was inverted and gently brushed the water surface to transfer the gathered nanosheets onto the substrate (Figure S1). The membrane was rinsed gently with deionized water to remove unreacted residual, dried at room temperature for 2 days, and stored in a desiccator for subsequent gas separation testing.

Gas separation tests

Gas separation performance of the prepared membranes was evaluated using the standard Wicke–Kallenbach method. For single gas permeation, the feed gases (H_2, CO_2, CH_4, C_2H_6, and C_3H_8) and the argon sweep gas were each maintained at a flow rate of 100 mL/min. For binary gas separation, an equimolar H_2/CO_2 mixture (a flow rate of 100 mL/min) was used, with the argon sweep gas also maintained at 100 mL/min. Both the feed and permeate sides were operated at atmospheric pressure (1 bar).

Temperature-dependent permeation was conducted by heating at a rate of 1 °C/min, with each temperature held for 120 min to ensure equilibrium.

Gas compositions in the permeate stream were analyzed using an Agilent 7890b gas chromatograph. The permeance of gas i (P_i, $mol\cdot m^{-2}\cdot s^{-1}\cdot Pa^{-1}$) was calculated using equation (1):

$$P_i = \frac{N_i}{A\Delta P_i} \quad (1)$$

where N_i is the permeate rate of gas i (mol s^{-1}), A is the effective membrane area (m^2), and ΔP_i is the transmembrane pressure difference of gas i (Pa). Permeance is also expressed in Gas Permeatiob Unit (GPU), where:

1 GPU = 3.35×10^{-10} $mol\cdot m^{-2}\cdot s^{-1}\cdot Pa^{-1}$ at standard temperature and pressure.

The separation factor (SF) for a gas pair i/j was calculated using equation (2):

$$\alpha_{ij} = \frac{y_i/y_j}{x_i/x_j} \quad (2)$$

where x and y represent the molar fractions of corresponding gases i and j on the feed and permeate, respectively.

The temperature dependence of gas permeance was analyzed using the Arrhenius equation (3):

$$P_i = A_0 exp\left(\frac{-E_{app}}{RT}\right) \quad (3)$$

where P_i is the permeance of gas i, A_0 is the pre-exponential factor, E_{app} is the apparent activation energy, R is the ideal gas constant (8.314 $J \cdot mol^{-1} \cdot K^{-1}$), and T (K) is the absolute temperature in Kelvin.

Characterizations

- **SEM and EDS:** Morphologies and elemental distributions of the nanosheets and membranes were determined using a JSM-7900F scanning electron microscope (JEOL Ltd., Japan) equipped with energy-dispersive X-ray spectroscopy.
- **TEM:** Nanosheet microstructures were examined with a HT7700 transmission electron microscope (HITACHI, Japan), operated at 100 kV.
- **AFM:** Thickness measurements of the nanosheets were obtained using a NanoWizard atomic force microscope (JPK Instruments, Germany).
- **ATR-FTIR:** Chemical structures of the nanosheets were analyzed using attenuated total reflection-Fourier transform infrared (ATR-FTIR) spectrometer (Nicolet 6700, Thermo Scientific Co.; Vertex 70, Bruker Co.). Samples were either dispersed in KBr powder (transparent in the mid-IR region) or coated onto silicon wafers.
- **Water contact angle (WCA):** Membrane hydrophilicity was measured with a contact angle analyzer (KRUSS DSA100, Germany).
- **XPS:** Elemental compositions and valence states on the membrane surface were analysed via an X-ray photoelectron spectrometer (ESCALAB250xi, Thermo Fisher Scientific, USA) with a monochromatic Al Kα source at 1486.6 eV. The C 1s peak at 284.8 eV was used as the binding energy reference.

Supplementary Supplementary Figures

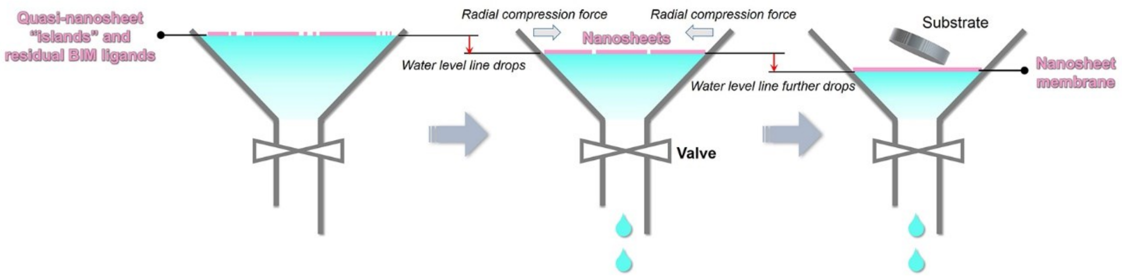

Supplementary Figure S1. Schematic illustration of the air–water interfacial synthesis process and sequential water-level drop/water surface constriction strategy used to fabricate continuous MOF nanosheet membranes in a custom-made conical funnel.

Note:

As a proof of concept, benzimidazole (BIM) ligands dissolved in toluene were dropwise applied onto an aqueous Zn^{2+} solution. Upon volatilization of toluene, the hydrophobic BIM ligands self-assembled at the air-water interface and coordinated with Zn^{2+} to form ultra-thin Zn-MOF nanosheets within 30 minutes at room temperature. A thin, colorful film resembling an oil layer was observed at the interface, indicative of nanosheet formation. The high surface-to-volume ratio of the reaction region facilitated rapid and generalized nanosheet synthesis. The resulting Zn-MOF nanosheets were assembled into continuous membranes using water-surface compression and Langmuir-Schaefer transfer technique.

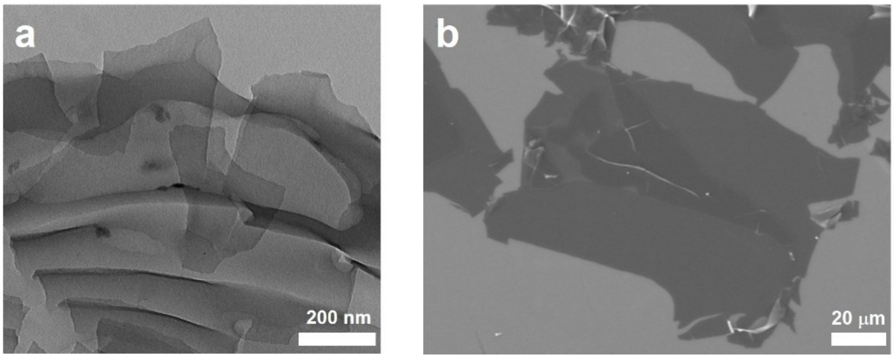

Supplementary Figure S2. (a) TEM and (b) SEM images of the synthesized Zn-MOF nanosheets.

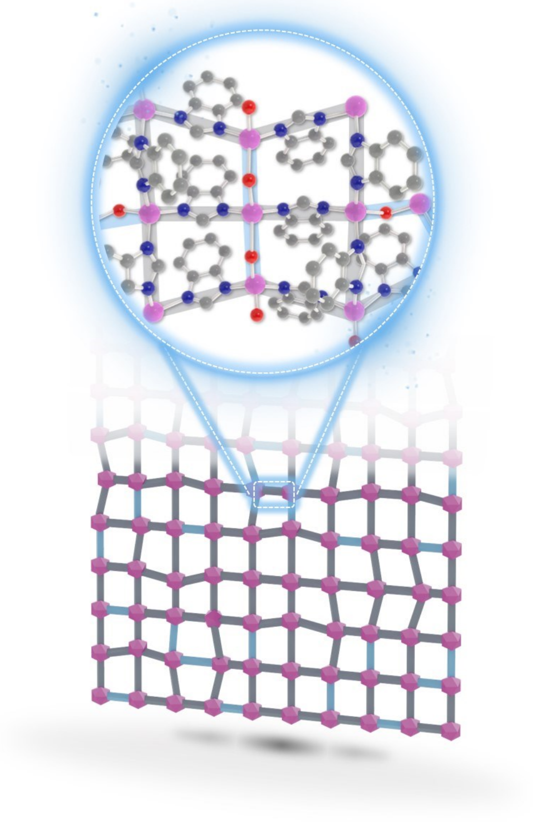

Supplementary Figure S3. Proposed microstructure of the Zn-MOF nanosheet. Pink: Zn atoms; red: O atoms; gray and blue: BIM ligand ring and water ligand; H atoms are omitted for clarity.

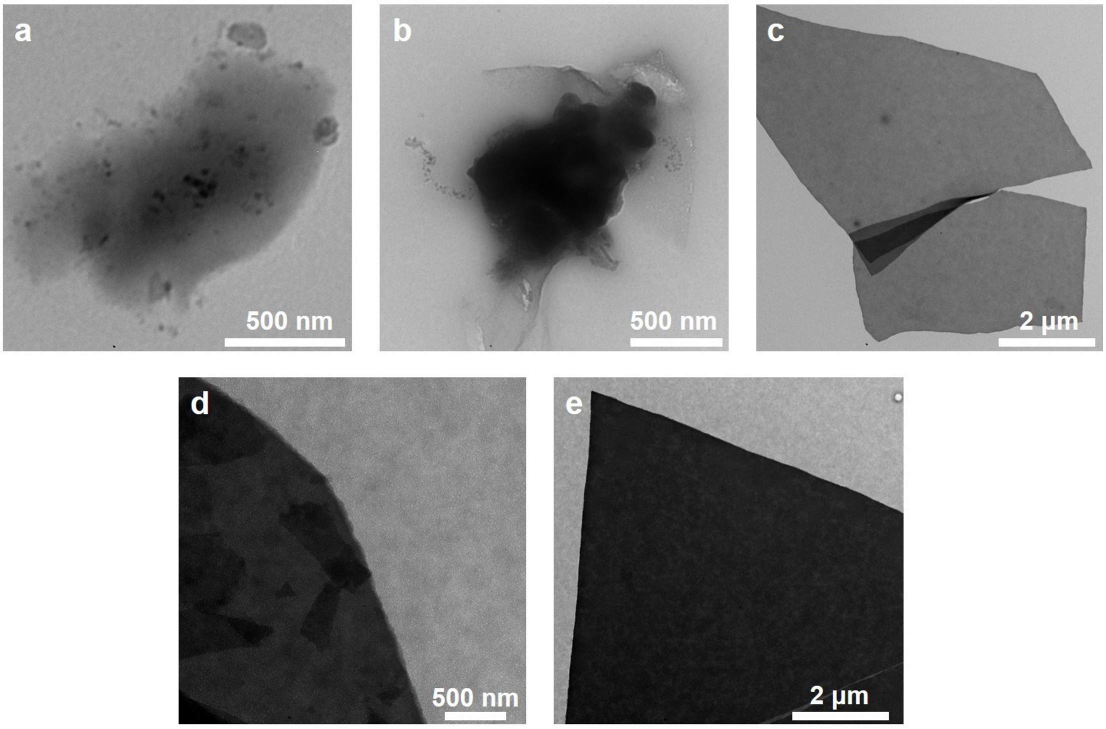

Supplementary Figure S4. TEM images of 2D Zn-MOF nanosheets synthesized using different volumes of BIM toluene solution: (a) 50 μL, (b) 100 μL, (c) 250 μL, (d) 500 μL, and (e) 1000 μL.

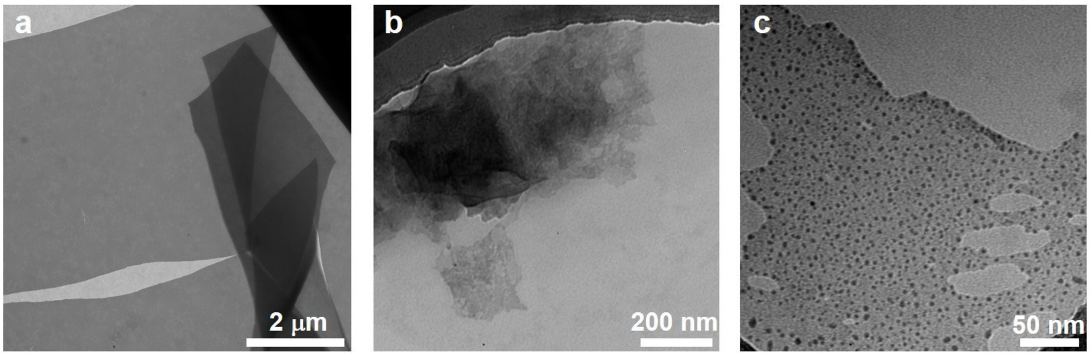

Supplementary Figure S5. TEM images of Zn-MOF samples: (a) with water-level drop and (b-c) without water-level drop.

The compaction effect induced by water-level lowering is evident in (a).

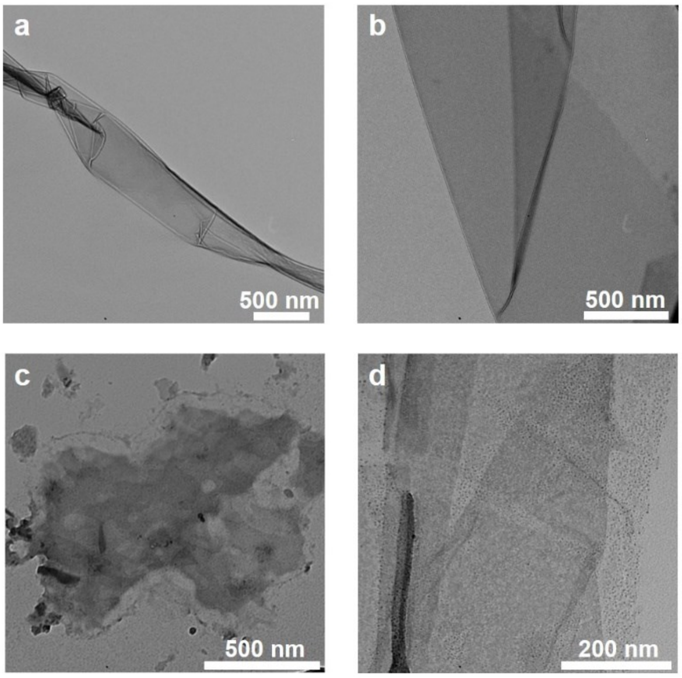

Supplementary Figure S6. TEM images of Zn-MOF nanosheets synthesized after different reaction durations prior to water-level drop: (a) 10 min, (b) 30 min, (c) 60 min, (d) 120 min. quasi-nanosheet islands and aggregated oligomers are clearly recognized in (d).

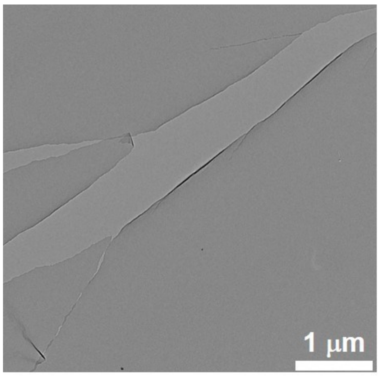

Supplementary Figure S7. TEM image of a 2D Zn-MOF nanosheet synthesized after 10 minutes of interfacial reaction.

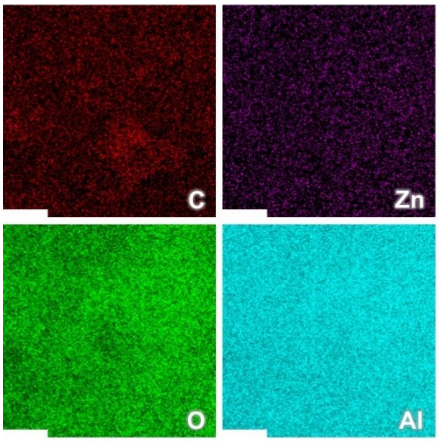

Supplementary Figure S8. Elemental mapping (EDS) of a 2D Zn-MOF nanosheet membrane. Scale bar: 1 μm.

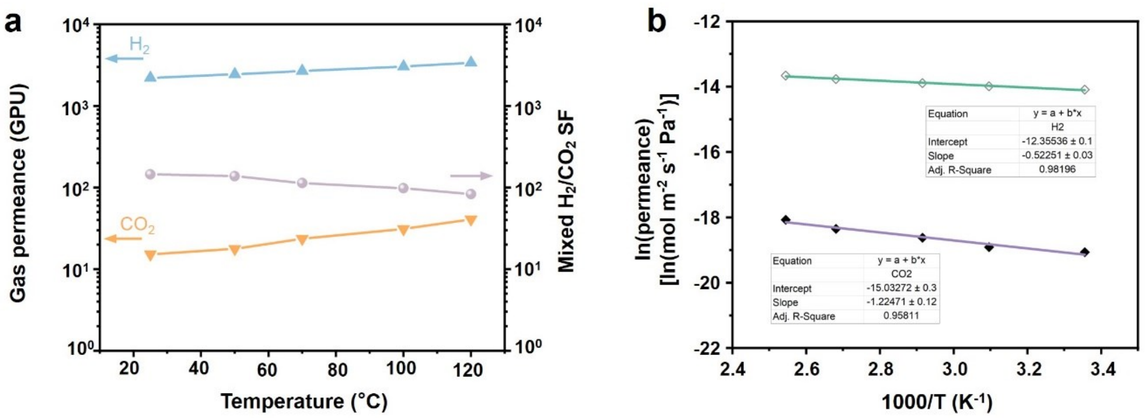

Supplementary Figure S9. (a) Temperature-dependent H_2/CO_2 separation performance of a Zn-MOF nanosheet membrane. (b) Corresponding Arrhenius plots for H_2 and CO_2 permeances.

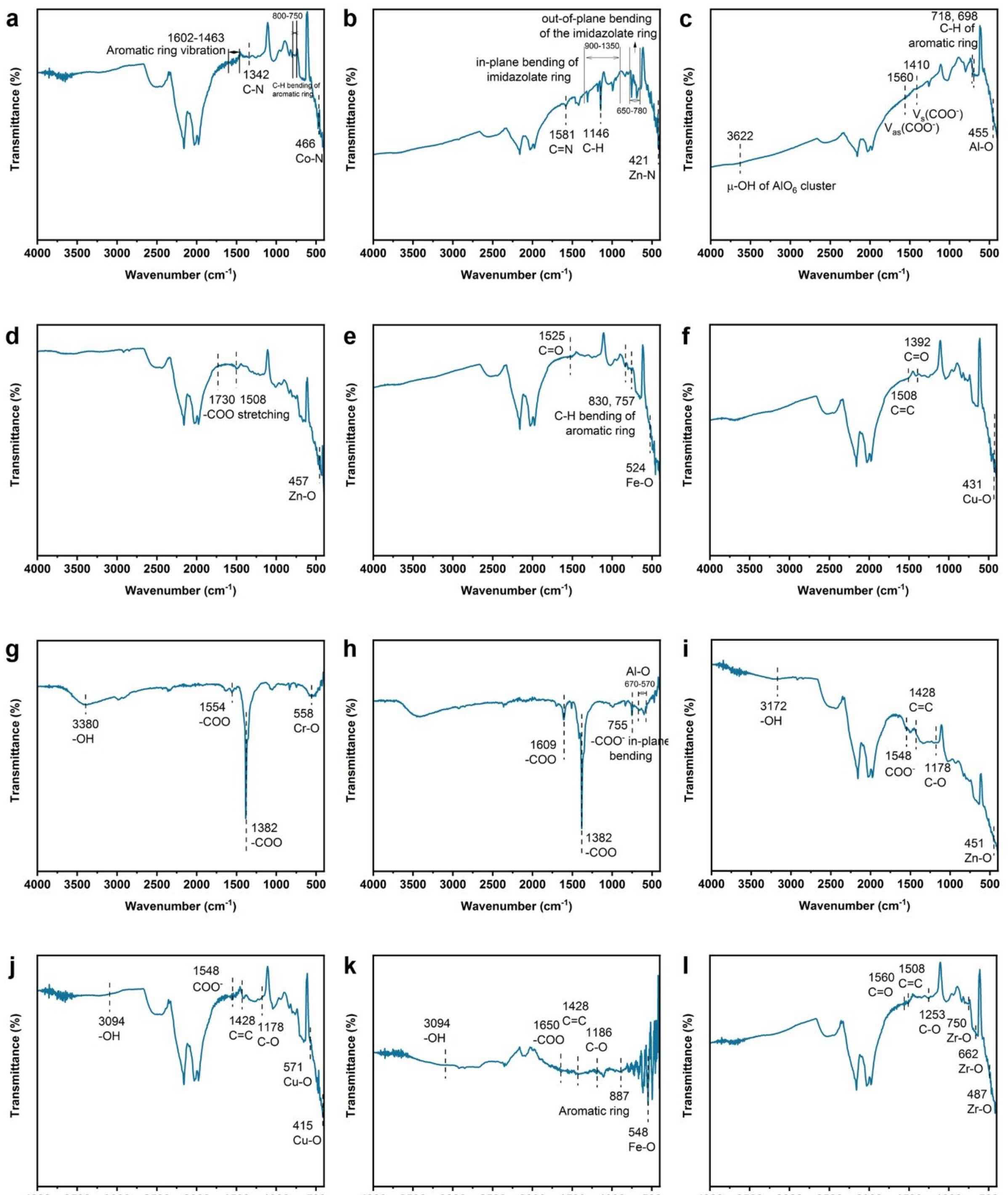

Supplementary Figure S10. FTIR spectra of 12 MOF nanosheet samples synthesized via the same interfacial synthesis strategy. Spectra (a) to (l) correspond to nanosheet samples (b-1) to (b-12) in **Figure 2** of the main manuscript. Spectra (g) and (h) were measured after dispersing the corresponding nanosheet samples in KBr powder which has no absorption in the mid-infrared region, all the others were coated on silicon wafers for analysis.

Supplementary Table S1. Comparison of fabrication conditions for reported 2D MOF nanosheet membranes.

membranes	Ligand*	Synthesis duration	Structure directing agent**	Ref.
$Zn_2(Bim)_4$	3.025 mg/mL BIM	**~13 d**	DEA	[1]
$[Cu_2Br(IN)_2]_n$	15 mg/mL INA	**~4 d**	/	[2]
Al-MOF	1 mg/mL H2TCPP	**~2 d**	pyrazine	[3]
PEI/NUS-8	2 mg/mL H3BTB	**~3 d**	formic acid	[4]
GO@Al-MOF	0.5 mg/mL H2TCPP	**>20 h**	pyrazine	[5]
NUS-8	1.76 mg/mL H3BTB	**~1 d**	formic acid	[6]
$Ni_3(HITP)_2$	1 mg/mL HITP	**>7 d**	/	[7]
$Cu(dhbc)_2(bpy)H_2O$	0.39 mg/mL 4,4'-bpy and 1.54 mg/mL H4DOBDC	**~4 d**	/	[8]
UiO-66-NH_2-P	3.1 mg/mL NH_2-BDC	**~5 d**	HAc	[9]
GO/Cu-TCPP	0.7 mg/mL H2TCPP	**>16 h**	BA	[10]
CuBDC	3.75 mg/mL H2BDC	**~3 d**	/	[11]
**This work**	**1 mg/mL BIM**	**~30 min**	**/**	**-**

*,** DEA: diethylamine, INA: isonicotinic acid, H2TCPP: meso-tetra(4-carboxyphenyl)porphine, H3BTB: 1,3,5-tri(4-carboxyphenyl)benzene, HITP: 2,3,6,7,10,11-hexaiminotriphenylene, 4,4'-bpy: 4,4′-bipyridine, H4DOBDC: 2,5-dihydroxyterephthalic acid, NH_2-BDC: 2-aminoterephthalic acid, HAc: acetic acid, BA: benzoic acid, H2BDC: terephthalic acid

Supplementary Table S2. Comparison of H_2/CO_2 separation performances between this work and previously reported membranes.

Category	Membrane	H_2 permeance (GPU)	Separation factor	Reference
MOF	Zn_2Bim_4	2700	291	[1]
	Zn_2Bim_3	2320	166	[12]
	ZIF-L	4033	321	[13]
	$(Zn/Co)_2(Bim)_4$	502	54.1	[14]
	ZIF-8	426	69.1	[15]
	ZIF-8/GO	406	25	[16]
	ZIF-8/GNR	1493	107	[17]
	ZIF-67@GO	518	140.9	[18]
	ZIF-95	567	184	[19]
	$[Cu_2Br(IN)_2]_n$	599	193	[2]
	N10-Zn_2Bim_4	1417	1158	[20]
	ZIF-67-in-TpPa-1	3253	33.3	[21]
	MAMS-1	714	245	[22]
	KAUST-7	1582	27.3	[23]
	CPM-5	3480	39.4	[24]
	CuBDC-GO	2866	95.1	[25]
	AO-PIM-1@ZIF-8	1671	12	[26]
	Co-gallate	150	60	[27]
	b-ZIF-L	1330	989	[28]
	NH_2-UiO-66	1230	41.3	[29]
	MIL-140A	705	598.6	[30]
	CuBDC-NO_2	1218	265.5	[31]
	ZIF-8	2653.7	17.1	[32]
COF	COF-300	2688	6	[33]
	ACOF-1	2018.9	14.1	[34]
	COF-LZU1-ACOF-1	660.2	24.2	
	TpPa-Me	727	12.7	[35]
	TpTGCl@TpPa-SO_3H/COF-LZ	2163	26	[36]

	Vertically aligned TFB-BD	3802.2	25.6	[37]
	Vertically aligned COF-LZU1	3654.8	31.6	
	N-COF	4319	13.8	[38]
	PVA@TpPa-SO_3H	1267.3	43	[39]
	TpEBr@TpPa-SO_3Na	2566	22.6	[40]
Zeolite	NaA	406	7.1	[41]
	ECNU-28	1272	61	[42]
	RUB-15	160	30	[43]
	LTA	4350	20.9	[44]
	ZSM-5-Silicate	371	25.3	[45]
	RUB-15	848	154.1	[46]
Polymer	CP glass	715	91	[47]
	m-PBI	48.5	33.3	[48]
	PBI-$TMA_{0.22}$	1.4	29	[49]
	PBDI	241	23	[50]
	BILP-101x	24.2	39.5	[51]
	MPD-TMC polyamide TFC	350	50	[52]
	T-C-BILP	257	38.5	[53]
	BILP-5	361.5	16.2	[54]
	PMDA-MA PI	9.1	18.2	[55]
	H_3PO_4/PBI	2.25	16	[56]
	BIALP	320	120	[57]
	2D Zn-MOF-dry gas-1	2117	196.5	**This work**
	2D Zn-MOF-dry gas-2	2462	114.5	
	2D Zn-MOF-dry gas-3	1949	183.7	
	2D Zn-MOF-humid gas-1	1289	417	
	2D Zn-MOF-humid gas-2	1378	357	
	2D Zn-MOF-humid gas-3	1199	239	

Supplementary References

[1] Y. Peng, Y. Li, Y. Ban, H. Jin, W. Jiao, X. Liu, W. Yang, *Science* **2014**, *346*, 1356.

[2] S. Song, W. Wang, Y. Zhao, W. Wu, Y. Wei, H. Wang, *Angew. Chem. Int. Ed.* **2023**, *62*, e202312995.

[3] M. Jian, R. Qiu, Y. Xia, J. Lu, Y. Chen, Q. Gu, R. Liu, C. Hu, J. Qu, H. Wang, X. Zhang, *Sci. Adv.* **2020**, *6*, eaay3998.

[4] Y. Pu, G. He, M. Zhao, Z. Yang, H. Li, Y. Ren, M. Long, X. Wang, D. Zhao, Z. Jiang, *J. Membr. Sci.* **2024**, *707*, 123018.

[5] H. G. Alemayehu, C. Liu, J. Hou, J. Yang, M. Fang, Z. Tang, L. Li, *J. Membr. Sci.* **2022**, *652*, 120479.

[6] H. Yuan, K. Li, D. Shi, H. Yang, X. Yu, W. Fan, P. J. S. Buenconsejo, D. Zhao, *Adv. Mater.* **2023**, *35*, 2211859.

[7] S. Jiang, X. Shi, F. Sun, G. Zhu, *Chem. Asian J.* **2020**, *15*, 2371.

[8] Y. Ying, Z. Zhang, S. B. Peh, A. Karmakar, Y. Cheng, J. Zhang, L. Xi, C. Boothroyd, Y. M. Lam, C. Zhong, D. Zhao, *Angew. Chem. Int. Ed.* **2021**, *60*, 11318.

[9] J. Xiao, M. Cong, M. Li, X. Zhang, Y. Zhang, X. Zhao, W. Lu, Z. Guo, X. Liang, G. Qing, *Adv. Func. Mater.* **2024**, *34*, 2307996.

[10] Z. Wang, J. Zhu, S. Xu, Y. Zhang, B. Van der Bruggen, *J. Membr. Sci.* **2021**, *633*, 119397.

[11] T. Rodenas, I. Luz, G. Prieto, B. Seoane, H. Miro, A. Corma, F. Kapteijn, F. X. Llabrés i Xamena, J. Gascon, *Nat. Mater.* **2015**, *14*, 48.

[12] Y. Peng, Y. Li, Y. Ban, W. Yang, *Angew. Chem. Int. Ed.* **2017**, *56*, 9757.

[13] K. Yang, S. Hu, Y. Ban, Y. Zhou, N. Cao, M. Zhao, Y. Xiao, W. Li, W. Yang, *Sci. Bull.* **2021**, *66*, 1869.

[14] C. Ma, G. Gao, H. Liu, Y. Liu, X. Zhang, *J. Membr. Sci.* **2022**, *644*, 120167.

[15] J. Hou, X. Hong, S. Zhou, Y. Wei, H. Wang, *AIChE J.* **2019**, *65*, 712.

[16] X. Wu, H. Zhang, Z. Yin, Y. Yang, Z. Wang, *J. Membr. Sci.* **2022**, *647*, 120291.

[17] E. Choi, J. I. Choi, Y.-J. Kim, Y. J. Kim, K. Eum, Y. Choi, O. Kwon, M. Kim, W. Choi, H. Ji, S. S. Jang, D. W. Kim, *Angew. Chem. Int. Ed.* **2022**, *61*, e202214269.

[18] Z. Li, Z. Li, N. Zhang, J. Bao, X. Zhang, G. He, C. Chen, Y. Song, *J. Membr. Sci.* **2024**, *694*, 122428.

[19] A. Deng, X. Shen, Z. Wan, Y. Li, S. Pang, X. He, J. Caro, A. Huang, *Angew. Chem. Int. Ed.* **2021**, *60*, 25463.

[20] H. Song, Y. Peng, C. Wang, L. Shu, C. Zhu, Y. Wang, H. He, W. Yang, *Angew. Chem. Int. Ed.* **2023**, *62*, e202218472.

[21] H. Fan, M. Peng, I. Strauss, A. Mundstock, H. Meng, J. Caro, *Nat. Commun.* **2021**, *12*, 38.

[22] X. Wang, C. Chi, K. Zhang, Y. Qian, K. M. Gupta, Z. Kang, J. Jiang, D. Zhao, *Nat. Commun.* **2017**, *8*, 14460.

[23] J. Lv, Y. Cui, J. Yang, L. Li, X. Zhou, J. Lu, G. He, *ACS Appl. Mater. Interfaces* **2022**, *14*, 4297.

[24] G. Yu, X. Shangguan, Z. Wang, H. Rong, K. Wang, Y. Zhang, T. Shao, X. Zou, *Inorg. Chem. Front.* **2022**, *9*, 1636.

[25] F. Yang, M. Wu, Y. Wang, S. Ashtiani, H. Jiang, *ACS Appl. Mater. Interfaces* **2019**, *11*, 990.

[26] S. Xiong, C. Pan, G. Dai, C. Liu, Z. Tan, C. Chen, S. Yang, X. Ruan, J. Tang, G. Yu, *J. Membr. Sci.* **2022**, *645*, 120217.

[27] Y. Liu, H. Chen, T. Li, Y. Ren, H. Wang, Z. Song, J. Li, Q. Zhao, J. Li, L. Li, *Angew. Chem. Int. Ed.* **2023**, *62*, e202309095.

[28] M. Zhao, D.-D. Zhou, P. Chen, Y. Ban, Y. Wang, Z. Hu, Y. Lu, M.-Y. Zhou, X.-M. Chen, W. Yang, *Sci. Adv.* **2023**, *9*, eadg2229.
[29] Y. Sun, J. Yan, Y. Gao, T. Ji, S. Chen, C. Wang, P. Lu, Y. Li, Y. Liu, *Angew. Chem. Int. Ed.* **2023**, *62*, e202216697.
[30] L. Liu, Y. Peng, K. Li, C. Zhu, W. Yang, *Adv. Func. Mater.* **2024**, *34*, 2404643.
[31] W. Wu, X. Cai, X. Yang, Y. Wei, L. Ding, L. Li, H. Wang, *Nat. Commun.* **2024**, *15*, 10730.
[32] Z. Gao, B. Li, Z. Li, T. Yu, S. Wang, Q. Fang, S. Qiu, M. Xue, *ACS Appl. Mater. Interfaces* **2023**, *15*, 19241.
[33] J. Fu, S. Das, G. Xing, T. Ben, V. Valtchev, S. Qiu, *J. Am. Chem. Soc.* **2016**, *138*, 7673.
[34] H. Fan, A. Mundstock, A. Feldhoff, A. Knebel, J. Gu, H. Meng, J. Caro, *J. Am. Chem. Soc.* **2018**, *140*, 10094.
[35] W. Zheng, J. Hou, C. Liu, P. Liu, L. Li, L. Chen, Z. Tang, *Chem. Asian J.* **2021**, *16*, 3624.
[36] Y. Ying, S. B. Peh, H. Yang, Z. Yang, D. Zhao, *Adv. Mater.* **2022**, *34*, 2104946.
[37] H. Fan, M. Peng, I. Strauss, A. Mundstock, H. Meng, J. Caro, *J. Am. Chem. Soc.* **2020**, *142*, 6872.
[38] X. Ma, N. Sun, Z. Li, M. Tong, Q. Ding, Z. Wang, L. Bai, L. Dong, Y. Liu, *Adv. Func. Mater.* **2024**, *34*, 2312203.
[39] X. Tian, L. Cao, K. Zhang, R. Zhang, X. Li, C. Yin, S. Wang, *Angew. Chem. Int. Ed.* **2025**, *64*, e202416864.
[40] Y. Ying, M. Tong, S. Ning, S. K. Ravi, S. B. Peh, S. C. Tan, S. J. Pennycook, D. Zhao, *J. Am. Chem. Soc.* **2020**, *142*, 4472.
[41] X.-L. Wei, W.-Y. Pan, M. Pan, L.-L. Peng, C.-Q. Niu, Z.-S. Chao, *J. Eur. Ceram. Soc.* **2020**, *40*, 1709.
[42] M. Dakhchoune, X. Duan, L. F. Villalobos, C. E. Avalos, K. V. Agrawal, *J. Membr. Sci.* **2023**, *672*, 121454.
[43] J. Wang, Y. Fan, J. Jiang, Z. Wan, S. Pang, Y. Guan, H. Xu, X. He, Y. Ma, A. Huang, P. Wu, *Angew. Chem. Int. Ed.* **2023**, *62*, e202304734.
[44] P. Yu, G. Yang, Y. Chai, L. Tosheva, C. Wang, H. Jiang, C. Liu, H. Guo, *Chem. Eng. J.* **2024**, *479*, 147629.
[45] H. Wang, Y. S. Lin, *J. Membr. Sci.* **2012**, *396*, 128.
[46] M. Dakhchoune, L. F. Villalobos, R. Semino, L. Liu, M. Rezaei, P. Schouwink, C. E. Avalos, P. Baade, V. Wood, Y. Han, M. Ceriotti, K. V. Agrawal, *Nat. Mater.* **2021**, *20*, 362.
[47] J. Li, J. Wang, Q. Li, M. Zhang, J. Li, C. Sun, S. Yuan, X. Feng, B. Wang, *Angew. Chem. Int. Ed.* **2021**, *60*, 21304.
[48] J. Sánchez-Laínez, M. Etxeberria-Benavides, O. David, C. Téllez, J. Coronas, *ChemSusChem* **2021**, *14*, 952.
[49] L. Hu, V. T. Bui, S. Fan, W. Guo, S. Pal, Y. Ding, H. Lin, *J. Mater. Chem. A* **2022**, *10*, 10872.
[50] M. Shan, X. Liu, X. Wang, Z. Liu, H. Iziyi, S. Ganapathy, J. Gascon, F. Kapteijn, *J. Mater. Chem. A* **2019**, *7*, 8929.
[51] M. Shan, X. Liu, X. Wang, I. Yarulina, B. Seoane, F. Kapteijn, J. Gascon, *Sci. Adv.* **2018**, *4*, eaau1698.
[52] Z. Ali, F. Pacheco, E. Litwiller, Y. Wang, Y. Han, I. Pinnau, *J. Mater. Chem. A* **2018**, *6*, 30.
[53] A. Gao, X. Yan, S. Cong, X. Wang, H. Liu, Z. Wang, X. Liu, *J. Membr. Sci.* **2023**, *668*, 121293.

[54] S. Duan, D. Li, X. Yang, C. Niu, S. Sun, X. He, M. Shan, Y. Zhang, *J. Membr. Sci.* **2023**, *671*, 121396.
[55] Z. Guo, S. Cong, L. Luan, M. Li, C. Luo, C. Wang, Z. Wang, X. Liu, *AIChE J.* **2023**, *69*, e18226.
[56] L. Zhu, M. T. Swihart, H. Lin, *Energ. Environ. Sci.* **2018**, *11*, 94.
[57] X. Yan, T. Song, M. Li, Z. Wang, X. Liu, *Nat. Commun.* **2024**, *15*, 628.
